# Change of Base

**Published:** 1867-12

**Authors:** 


***Changed his Base.***

The following notice taken from a Chicago paper referring to an individual who lately operated on eyes, ears and pockets in San Francisco, will be interesting to a large circle of tradespeople in this city:—We notice the return of Dr. De Castro to Chicago, to his former residence, 385 Wabash Avenue. The success that attended the Doctor's treatment of disease of the head, throat and lungs last Winter, established for him a large practice in this city, in consequence of which he has returned to resume his duties.—*Pacific Med. & Surg. Journal.*

